# Knowledge, attitudes, and practices related to bovine tuberculosis in Benin: a mixed One Health survey among farmers, veterinarians, and butchers

**DOI:** 10.1016/j.onehlt.2026.101577

**Published:** 2026-09-12

**Authors:** Nongande Pioulasse Bernice Houecande, Tamégnon Victorien Dougnon, Roch Houngnihin, Souaïbou Farougou, Kadoéito Cyrille Boko, Claude Saegerman

**Affiliations:** a Research Unit on Communicable Diseases, Polytechnic School of Abomey-Calavi (EPAC), University of Abomey-Calavi (UAC), Abomey-Calavi, Benin; b Research Unit in Applied Microbiology and Pharmacology of Natural Substances, Polytechnic School of Abomey-Calavi (EPAC), University of Abomey-Calavi (UAC), Abomey-Calavi, Benin; c Laboratory of Applied Medical Anthropology (LAMA), University of Abomey-Calavi (UAC), Abomey-Calavi, Benin; d Fundamental and Applied Research for Animal and Health (FARAH) Center, University of Liege, Liege, Belgium

**Keywords:** Bovine tuberculosis, *Mycobacterium bovis*, One Health, KAP, Pastoral system, Benin

## Abstract

Bovine tuberculosis (bTB), caused by *Mycobacterium bovis*, is an underrecognized zoonotic disease in sub-Saharan Africa affecting health, food security, and livelihoods. In Benin, research mainly detects it in animal products and carcasses, but behavioral and sociocultural factors related to exposure and transmission are poorly documented. In areas with livestock farming, pastoral mobility, human-animal interactions, and unsafe animal product consumption, assessing knowledge, attitudes, and practices (KAP) is crucial for understanding transmission within a One Health approach. This study aimed to assess the knowledge, attitudes, and practices related to bovine tuberculosis among livestock farmers, veterinarians, and butchers/slaughterhouse workers in Benin's Agricultural Development Pole 4 (PDA4), while integrating quantitative and qualitative findings within a One Health framework. A mixed-methods cross-sectional survey was conducted among livestock farmers, veterinarians, and slaughterhouse workers across seven municipalities in PDA4. Quantitative data from questionnaires and qualitative data were analyzed thematically. Among livestock farmers (*n* = 164), knowledge was low (41.6%), attitudes favorable (80.0%), and practices moderate (51.5%). A positive correlation existed between knowledge and attitudes (ρ = 0.42; *p* < 0.001), but no link between knowledge and practices. Veterinarians (*n* = 36) had high knowledge (80.0%), but lower attitudes (42.2%) and practices (44.8%), with a negative correlation between knowledge and attitudes (ρ = −0.385; *p* = 0.02). Among butchers (*n* = 11), knowledge was intermediate (63.64%), attitudes very favorable (96.97%), and practices satisfactory (69.09%), with no significant correlations. Qualitative findings identified key behavioral factors like raw milk consumption, traditional practices, economic constraints, and non-biomedical disease perceptions. Major risk factors included close contact, shared water points, and transboundary herd movement. Knowledge of bTB is limited in surveyed departments. Territorial differences influence practices and risks; two communes are more vulnerable because of intensive pastoralism and shared watering points. Knowledge gaps reflect behaviors shaped by awareness, social, economic, cultural, and environmental factors. The study emphasizes tailored, culturally sensitive control strategies within a One Health approach. These findings should be interpreted considering the cross-sectional design, which precludes causal inference, and the limited number of butchers surveyed, which restricts the statistical power of subgroup analyses.

## Highlights

• Livestock farmers in Benin possessed only limited knowledge of bovine tuberculosis.
• Human-animal interactions and unsafe practices can facilitate the transmission of *Mycobacterium bovis*.
• Shared water sources and pastoral mobility create additional opportunities for disease spread.
• There were notable differences across regions regarding exposure and risk factors.
• Merely being aware of the disease did not reliably indicate whether farmers engaged in preventive practices.
• Economic, cultural, and environmental influences played a major role in shaping protective behaviors.
• Social and cultural factors also affected risk-related actions.
• Adopting a One Health approach is essential for enhancing the management of bovine tuberculosis.

## Graphical abstract

**Figure:**
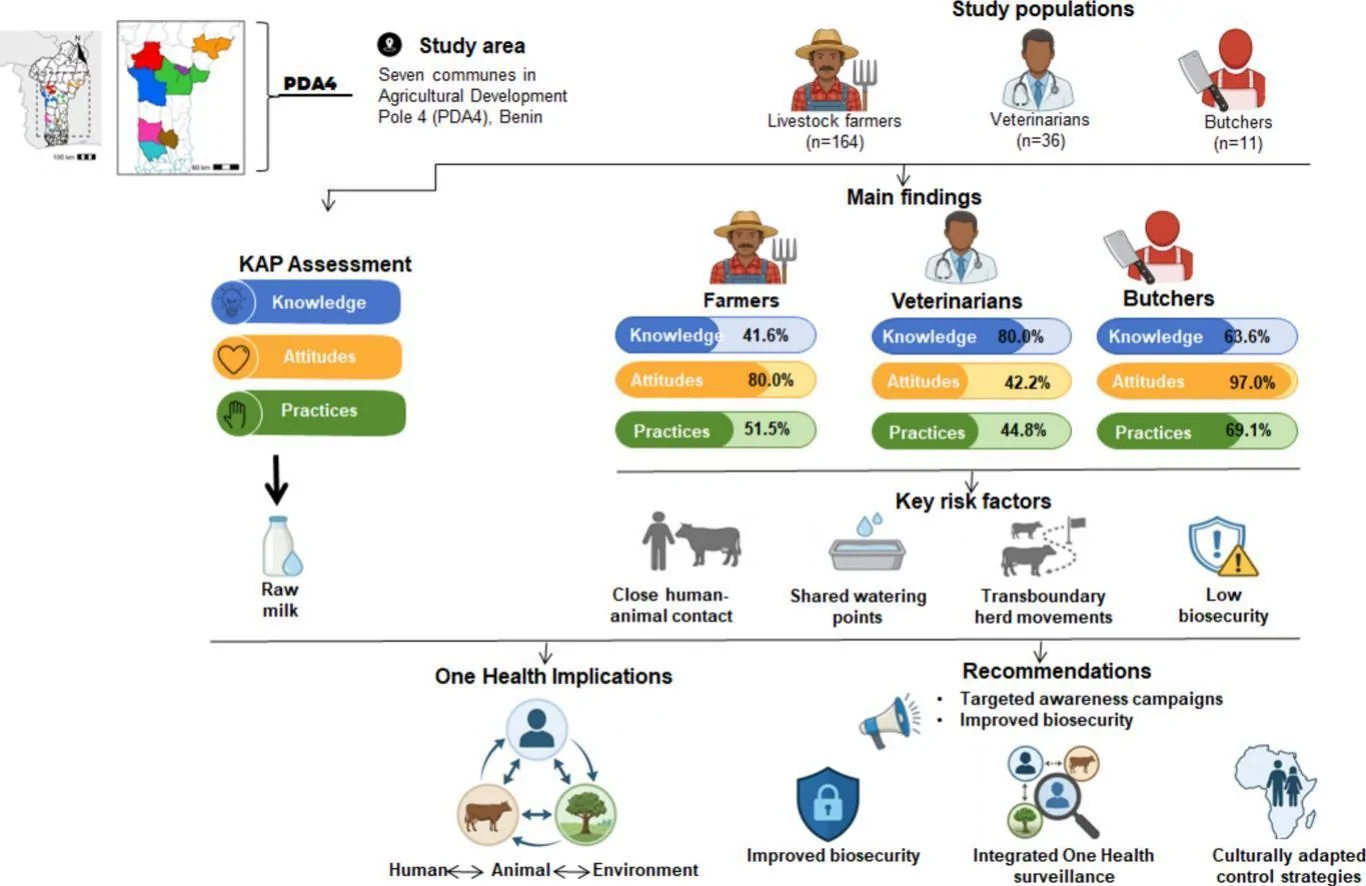

## 1 Introduction

Bovine tuberculosis (bTB) caused by *Mycobacterium bovis* (*M. bovis*), currently classified in the NCBI Taxonomy as *Mycobacterium tuberculosis* variant *bovis*, is a chronic infection at the interface of animal, human, and environmental health [1]. It primarily affects cattle but can also infect other animal species and humans, particularly through the consumption of unpasteurized milk or contaminated animal products, or by inhaling infectious aerosols during close contact with animals or their carcasses [2], [3], [4], [5]. Globally, bTB remains widely distributed, although its epidemiological burden varies considerably by region, depending on surveillance capacity, livestock production systems, diagnostic methods, and the implementation of prevention and control programs. Despite substantial progress in controlling the disease in several high-income countries, bTB remains endemic in many low- and middle-income countries, where limitations in surveillance and prevention and control contribute to persistent animal and zoonotic transmission [6], [7], [8], [9].In sub-Saharan Africa, the epidemiological burden remains substantial but heterogeneous.

Globally, zoonotic tuberculosis caused by *M. bovis* is estimated to account for approximately 140,000 human cases and 11,400 deaths annually worldwide, around 1.4% of the global tuberculosis burden, with the highest case numbers reported from Africa and Southeast Asia. In sub-Saharan Africa, a recent meta-analysis estimated the pooled prevalence of tuberculosis in cattle at 5.06%, with a higher burden in West Africa, while *M. bovis* prevalence among incident human tuberculosis cases reached 1.56%, particularly in West and East Africa [10]. The same study estimated the prevalence of *M. bovis* infection in humans at 0.73% (95% CI: 0.53–1.01) overall and 1.56% (95% CI: 1.04–2.33) among incident tuberculosis cases, with higher prevalence reported among livestock workers. These findings highlight the persistence of zoonotic transmission at the human-animal interface, particularly in settings characterized by close livestock contact and limited surveillance capacity. In West Africa, epidemiological estimates vary markedly between countries and study settings. Available evidence indicates cattle prevalence ranging from below 1% in some abattoir-based studies to above 10% in specific cattle populations, while substantial heterogeneity in diagnostic methods and sampling strategies limits direct comparisons. This variability is particularly relevant in extensive and transhumant livestock systems, where herd mobility, communal grazing, shared watering points, close human-animal contact, and cross-border livestock movements may facilitate the maintenance and geographical spread of *M. bovis* [11], [12], [13], [14]*.* In Benin, livestock farming is a key agricultural sector and an important source of subsistence. Approximately 40% of the national cattle herd is concentrated in the Agricultural Development Pole 4 (PDA_4_) area [15], [16], [17], [18], underscoring the importance of this area for livestock health and production. Available epidemiological evidence confirms the occurrence of bTB in cattle and the presence of *M. bovis* at the livestock food interface. In Benin specifically, individual-animal tuberculosis prevalence was estimated at 2.18% in the country's main dairy basins, with marked regional variation [19], consistent with earlier reports of *M. bovis* detection in milk and meat in the Borgou department. More recently, molecular investigation of 40 tissue samples collected from ruminant carcasses with lesions suspected of bTB during routine meat inspection in Cotonou found 26 samples positive by GeneXpert (65%) [20]. This proportion reflects positivity among preselected suspected lesions and should therefore not be interpreted as population-level prevalence. Although these apparent prevalence estimates remain moderate compared with some other sub-Saharan settings, they likely underestimate the true burden given limited diagnostic coverage, and they do not capture the intensity of human-animal contact described below, which shapes the actual zoonotic risk. However, existing studies are fragmented, and documentation on the behavioral and socio-cultural factors influencing exposure among key occupational groups remains limited.

Beyond these epidemiological findings, understanding the problem remains incomplete without considering actors' daily behaviors, local beliefs, economic constraints that influence health decisions, and ecological interfaces such as water points or transhumance corridors [5], [21]. For most at-risk populations living in these contexts, where the disease is out of control and where traditional screening and elimination programs are socio-economically unfeasible [22], it is crucial to better understand social behaviors in the face of zoonotic tuberculosis.

International literature on KAP surveys emphasizes that understanding knowledge, attitudes, and practices is essential for developing awareness-raising, communication, and behavior change actions based on reliable data [9]. However, several studies also show that improved knowledge does not automatically guarantee the adoption of better practices, especially when these behaviors are rooted in cultural norms, material constraints, or professional routines [14], [23]. In pastoral systems, health decisions often occur at the intersection of lay knowledge, technical knowledge, household economics, and relationships of trust or mistrust toward institutions [24], [25]. Participatory epidemiology approaches also highlight this complexity, recognizing that livestock farmers possess empirical knowledge about diseases and actively participate in their management. This implies that control strategies must be co-developed with communities [26].

Beyond its health implications, bTB has significant socio-economic consequences in sub-Saharan Africa, where livestock constitutes a critical source of income, nutrition, social status, and household resilience. The disease reduces milk production, limits trade, and increases animal mortality and management costs, thereby directly affecting the livelihoods of livestock farmers [27]^.^ According to the Directorate of Agricultural Statistics [18], annual losses in Benin's dairy sector are estimated at approximately US$2.5 million. For individual farmers, treating an infected animal may cost around US$120, which can represent nearly three months of income for small-scale producers.

Within this context, farmers interact directly with animals, water sources, animal products, and the domestic environment; veterinarians contribute technical knowledge, surveillance, and preventive messages; and butchers are exposed to carcasses, potentially infected tissues, and occupational and commercial constraints.

This survey was designed using a One Health approach. Its objective is to analyze the knowledge, attitudes, and practices regarding bTB among livestock farmers, veterinarians, and butchers/slaughterhouse workers in the PDA_4_ region, integrating quantitative and qualitative findings. More specifically, it aims to: (i) describe the socio-demographic and professional profiles of the participants; (ii) measure their levels of knowledge, attitudes, and practices; (iii) analyze the relationships between these dimensions; (iv) interpret the results based on qualitative data; and (v) discuss these results at the municipal, departmental, and PDA_4_ levels as a whole, in order to provide a coherent understanding of behavioral and environmental vulnerabilities from a One Health perspective.

## 2 Materials and methods

### 2.1 Survey area and framework

**Fig. 1:**
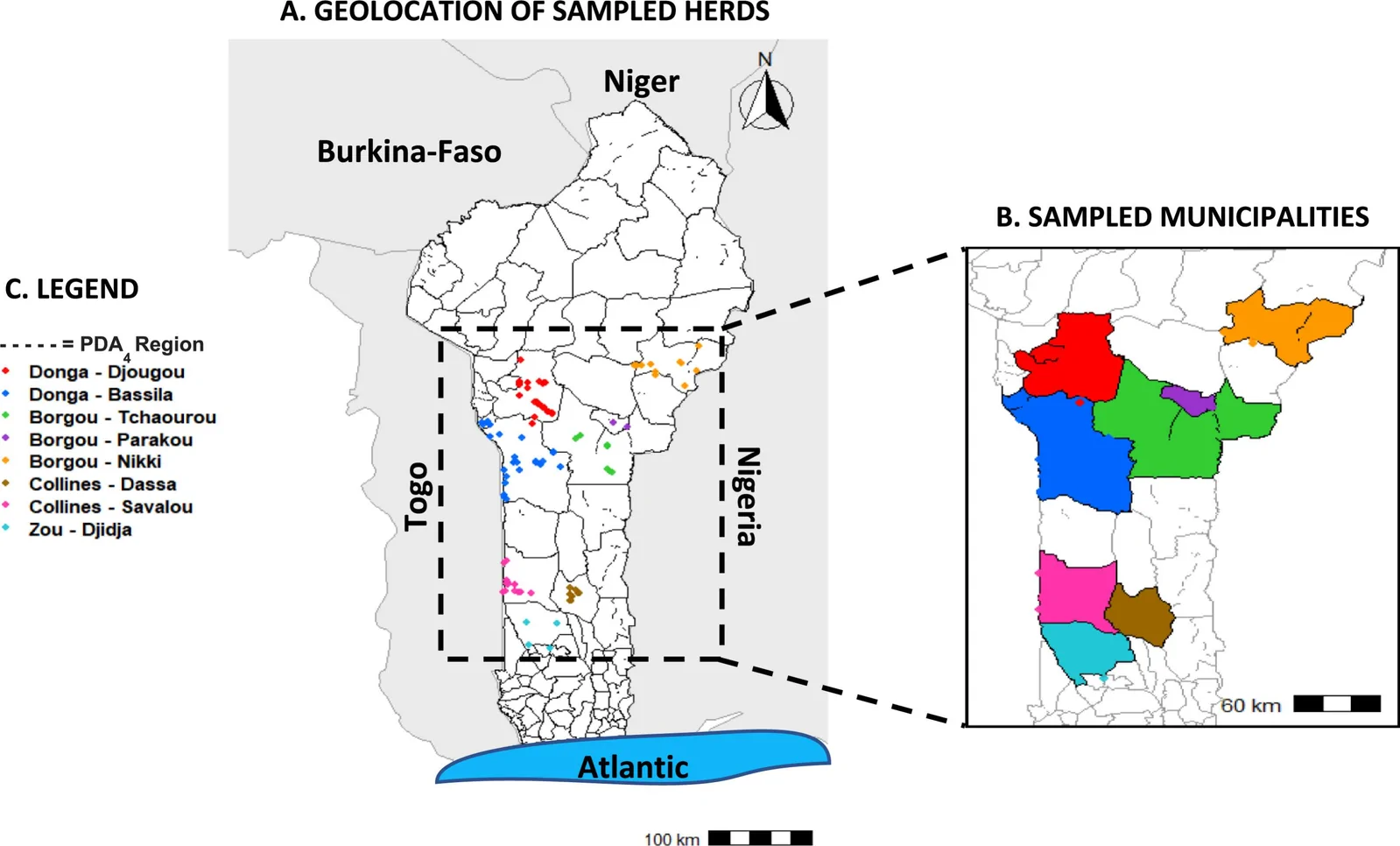
Location of the study municipalities and surveyed cattle production areas in Agricultural Development Pole 4 (PDA4), Benin. Bassila and Djougou (Donga Department), Tchaourou and Nikki (Borgou Department), Dassa-Zoumè and Savalou (Collines Department), and Djidja (Zou Department). Colored points indicate surveyed localities, while the inset map highlights the municipalities included in the study.

The study was conducted in Agricultural Development Pole 4 (PDA4), located in central Benin and commonly referred to as the Borgou South-Donga-Collines agricultural development zone. The area extends approximately between 7°00′ and 10°40′ N and 1°25′ and 3°50′ E and encompasses a large and ecologically diverse portion of central Benin. PDA4 includes municipalities distributed mainly across the departments of Borgou, Donga, and Collines, with Djidja municipality in the Zou Department also included in the agricultural development pole. The region is characterized by a Sudano-Guinean transitional climate, with annual rainfall generally ranging from approximately 900 to 1.110 mm and mean temperatures of 25–29 °C. Its vegetation consists predominantly of savannah, woodland, fallow areas, dry forests, and riparian vegetation, providing diverse grazing and agricultural resources. PDA4 has a strongly integrated agro-sylvo-pastoral production system. Crop production includes cotton, cashew, maize, rice, soybean, cowpea, groundnut, cassava, yam, and mango, while livestock farming is an important component of local livelihoods. Cattle, sheep, goats, and poultry are widely raised, and the pole is recognized among the major livestock-producing areas of Benin. Recent livestock data indicate substantial cattle populations in several municipalities, including Tchaourou, Djougou, Nikki, Savalou, Djidja, and Bassila (Fig. 1).

Regarding cattle production, the data from the Directorate of Agricultural Statistics (DSA) show that, PDA4 had 868,858 cattle in 2023, accounting for 34.37% of the national total, making it the second-largest cattle region after PDA2. Borgou South alone had 455,337 cattle, followed by Collines with 238,994, Donga with 180,377, and Djidja with 51,089 [18]. These municipalities in Table 1 feature high cattle densities, large livestock systems, seasonal transhumance, active markets, and interactions among farmers, butchers, veterinary staff, and consumers. These factors facilitate the spread of bovine tuberculosis and other zoonoses, emphasizing PDA4's role in assessing knowledge, attitudes, and practices about bovine tuberculosis within a One Health framework.

**Table 1:** Summary of investigated variables according to actor categories in the One Health KAP survey on bovine tuberculosis in PDA4, Benin.

Department	Number of cattle
PDA4	868,858
Sud Borgou	455,337
Tchaourou	125,081
Nikki	102,806
Ndali	86,161
Perèrè	71,859
Parakou	12,491
Collines	238,994
Savalou	61,637
Dassa	42,085
Ouesse	40,130
Save	37,369
Glazoue	34,476
Bante	23,297
Donga	180,377
Djougou	79,969
Bassila	52,523
Ouake	29,936
Copargo	17,949
Zou	51,089
Djidja	51,089

### 2.2 Type of survey

This was a mixed-methods cross-sectional survey that combined a quantitative KAP component with a qualitative component based on interviews and their analytical transcription. This methodological choice responded to the need to avoid reducing bTB to a simple technical or diagnostic problem, while also understanding the representations, constraints, and practical logics of the actors involved [28], [29], [30].

### 2.3 Survey population, sampling, and recruitment

#### 2.3.1 Survey population

The reference population of the survey comprised three key stakeholder groups involved in the transmission and control of bovine tuberculosis (bTB): livestock farmers, veterinary personnel involved in animal health and/or meat inspection, and butchers/slaughterhouse workers, all professionally active within the seven selected municipalities of Agricultural Development Pole 4 (PDA4). Their inclusion was grounded in a One Health approach recognizing the interactions among humans, animals, and the environment, together with their direct and recurrent occupational contact with cattle, animal products, carcasses, or potentially infected tissues.

Livestock farmers were the main focus because of their daily contact with cattle and their central role in herd management, animal health practices, animal movements, and the production and consumption of animal products. Husbandry activities such as animal purchases, transhumance, herd mixing, sick-animal management, and raw-milk consumption can directly affect the persistence and spread of *Mycobacterium bovis* in cattle and at the human-animal interface. Veterinary staff were included because they represent the technical and institutional dimension of bTB surveillance and control, including disease detection, prevention, health monitoring, slaughterhouse inspections, awareness campaigns, and communication with farmers; as frontline professionals, they contribute to early case detection and the implementation of prevention and control measures. Butchers and slaughterhouse workers were selected because they operate at the animal-food-human interface, handling carcasses, blood, organs, and potentially tuberculous lesions, which may expose them to infection; their knowledge of carcass inspection, hygiene, safety measures, and personal protection is crucial for preventing zoonotic transmission and safeguarding food safety.

#### 2.3.2 Eligibility criteria

Eligible participants were individuals actively engaged in one of the three targeted occupational groups within the selected survey area who provided informed consent to participate. Individuals who did not belong to the targeted stakeholder groups, worked outside the selected survey area, declined to participate, or did not complete the questionnaire sufficiently for analysis were excluded.

#### 2.3.3 Sampling, recruitment and sample size

Recruitment took place within the municipalities and localities selected as part of the broader research protocol, using a non-probability, field-based (convenience/purposive) approach rather than random sampling from a formal sampling frame. Livestock farmers were recruited from selected cattle herds and livestock markets within these localities; eligible farmers encountered during field visits were invited to participate irrespective of their prior knowledge of bovine tuberculosis. Recruitment of the other stakeholder groups was adapted to their accessibility and professional organization. Veterinary personnel constituted a comparatively small and geographically dispersed professional group in the study area; their recruitment was facilitated with the support of the Departmental Union of Professional Livestock Breeders' Organizations (UDOPER) during professional gatherings held at the Departmental Directorates of Agriculture, Livestock and Fisheries (DDAEP), with particular representation from personnel involved in meat inspection. Butchers/slaughterhouse workers were recruited through a similar field-based approach among eligible workers accessible at slaughter and meat-processing sites during the data-collection period.

The broader research protocol included a priori sample-size calculations for its animal or biological component; however, this calculation was not used to determine the sample size of the present KAP survey. For the KAP component, participants were recruited within the predefined study localities based on eligibility and field accessibility, while recruitment of the less numerous professional groups, particularly veterinary staff and butchers / slaughterhouse workers, also depended on their availability during the survey period. The final analytical sample comprised 211 participants: 164 livestock farmers, 36 veterinary staff, and 11 butchers/slaughterhouse workers. Because the number of eligible individuals approached and those who declined to participate was not systematically recorded during field recruitment, a formal response rate could not be calculated, this is acknowledged as a limitation in the discussion.

### 2.4 Questionnaire development, pretesting, and administration

The doctoral researcher developed the structured questionnaires specifically for this study, with input from a sociologist and reference to previously published KAP surveys on bovine tuberculosis and related zoonotic disease risks. Separate stakeholder-specific versions were developed for livestock farmers, veterinary staff, and butchers/slaughterhouse workers to account for differences in occupational activities and exposure contexts, while maintaining a common framework covering knowledge, attitudes, and practices.

Before the main survey, the questionnaires were pre-tested with approximately 15 individuals from populations similar to the target groups. The pre-test assessed the clarity, comprehension, relevance, and practical administration of the questions before survey refining and field deployment. Minor wording and sequencing adjustments were made to improve clarity and facilitate field administration.

The doctoral researcher and three trained Master's students collected data through face-to-face interviews (conversation). Before data collection, the Master's students received training on the study objectives, questionnaire content, standardized interviewing procedures, informed consent, and accurate recording of participants' responses. Veterinary field guides familiar with the survey communities accompanied the research team and provided linguistic mediation when required. The questionnaires and final recording of responses were in French. For participants who were not sufficiently fluent in French, questions and responses were orally interpreted in the participant's preferred local language, mainly Fulbe (Peuhl), Fon, or Bariba, with the assistance of the veterinary field guides. The same general interview and data-recording procedures were applied across the survey sites.

### 2.5 Variables studied

The list of all variables studied is presented in Table 2, including department, municipality, sex, age, age group, religion, ethnic group, marital status, number of children, education level, approximate monthly income, and duration in the activity. For livestock farmers, additional variables were included: type of livestock farming, herd size, number of watering points, and motivations for choosing the activity. KAP scores were constructed from items specific to each of the three groups. The coding grid included in the qualitative and quantitative analysis documents details the variables used to calculate the scores.

**Table 2:** Summary of investigated variables according to actor categories in the One Health KAP survey on bovine tuberculosis in PDA4, Benin.

Actor category	Variable domain	Variables investigated
All participants	Socio-demographic characteristics	Department, municipality, sex, age, age group, marital status, religion, ethnic group, education level, approximate monthly income, years of professional experience/seniority
Livestock farmers	Livestock production system	Type of livestock farming system, herd size, grazing practices, transhumance practices, watering points used (rivers, marshes, dams, gullies), motivations for livestock farming (family inheritance, economic reasons, tradition)
	Knowledge variables	Awareness of bovine tuberculosis (bTB), local names of the disease, knowledge of zoonotic transmission, recognition of clinical signs, awareness of transmission routes, knowledge of preventive measures, participation in awareness campaigns
	Attitude variables	Perceived severity of bTB, perceived zoonotic risk, willingness to adopt preventive measures, confidence in veterinary recommendations, perception of awareness campaigns, willingness to report suspicious cases
	Practice variables	Raw milk consumption, milk boiling practices, management of sick animals, sale or slaughter of diseased animals, use of medicinal plants, veterinary consultation practices, carcass handling practices, isolation of sick animals, hygiene measures during animal handling
	Environmental exposure variables	Shared watering points, herd mixing, proximity between livestock and households, use of communal grazing areas, and environmental exposure during the dry season
Veterinary staff	Professional characteristics	Type of veterinary activity, years of professional experience, areas of intervention, involvement in surveillance activities, participation in awareness campaigns
	Knowledge variables	Knowledge of zoonotic tuberculosis, knowledge of transmission pathways, knowledge of clinical signs and lesions, understanding of surveillance systems, awareness of occupational exposure risks
	Attitude variables	Perception of disease importance, confidence in current control measures, perception of institutional support, attitudes toward surveillance implementation, perception of community collaboration
	Practice variables	Slaughterhouse inspection practices, use of personal protective equipment (PPE), carcass inspection procedures, disinfection practices, management of suspected cases, awareness and communication activities
Butchers and slaughterhouse workers	Professional characteristics	Years of professional experience, slaughtering activities, and carcass handling responsibilities
	Knowledge variables	Recognition of suspicious lesions, awareness of zoonotic transmission, and awareness of occupational exposure risks
	Attitude variables	Acceptance of sanitary seizure measures, perception of occupational risk, and willingness to report suspicious carcasses
	Practice variables	Use of Personal Protective Equipment (PPE), carcass handling practices, meat inspection practices, disposal of suspicious carcasses, cleaning and hygiene procedures
Qualitative and sociocultural variables	Sociocultural perceptions and practices	Local disease terminology, traditional beliefs related to bTB, perceptions of transmission, use of traditional medicine, trust in veterinary services, role of community leaders and local radio, cultural importance of raw milk consumption
One Health and environmental interface variables	Human-animal-environment interactions	Animal mobility, shared environmental resources, contact between humans and livestock, contamination risks associated with water, soil, forage, slaughter environments, and pastoral ecosystems

### 2.6 Construction of Knowledge, Attitudes, and Practices scores

Knowledge (K), attitude (A), and practice (P) scores were derived from responses to structured questionnaires, with each item assigned a score based on the accuracy or relevance of the responses, in accordance with standardized KAP survey approaches [23], [31].

The scoring system was based on composite variables derived from several indicators, in accordance with classic KAP survey approaches relating to:

▪ **Knowledge (K)**: recognition of the disease, clinical signs, modes of transmission, understanding of zoonosis.
▪ **Attitudes (A)**: perception of severity, perception of risk, acceptability of health measures.
▪ **Practices (P)**: health behaviors, dietary practices, biosecurity measures.

The overall scores for each dimension (K, A, P) were then standardized on a scale of 0 to 100 to enable comparability across actor groups and analytical dimensions. The overall formula for the knowledge (K), attitude (A) or Practices (P) scores calculated for each actor surveyed are:

(1) $$K\;\mathrm{score}=\sum_{i=1}^{n}K_{i}x\;100$$

(2) $$A\;\mathrm{score}=\sum_{i=1}^{n}A_{i}x\;100$$

(3) $$P\;\mathrm{score}=\sum_{i=1}^{n}P_{i}x\;100$$

With: K = Knowledge, A = Attitudes, and P = Practices.

The maximum scores were:

▪ For breeders: K = 7; A = 5; *P* = 9.
▪ For veterinarians: K = 7; A = 5; *P* = 8.
▪ For butchers: K = 5; A = 3; *P* = 5.

The scores obtained were presented using descriptive statistics (mean, median, quartiles, minimum, maximum) to characterize the distribution of KAP levels within the different groups studied.

### 2.7 Statistical analysis

The statistical analysis was performed using descriptive and inferential methods adapted to the nature of the data. The normality of the distributions of Knowledge, Attitudes and Practices (KAP) scores was evaluated using the Shapiro-Wilk test, recommended for moderately sized samples [32]. The threshold for statistical significance was set at *p* < 0.05. The results showed that the distributions of the knowledge, attitudes, and practices scores did not follow a normal distribution (*p* < 0.05). Consequently, Spearman's rank correlation coefficient was used to analyze the relationships among the KAP dimensions [33].

### 2.8 Qualitative analysis

The qualitative analysis was carried out using full interview transcripts, following an inductive thematic approach inspired by content analysis methods [34]. The data were coded and then organized into analytical categories structured around several key dimensions, including: local representations of bTB, care practices, feeding behaviors, methods of managing sick animals, forms of environmental exposure, herd mobility, human-animal proximity, and awareness channels deemed credible by stakeholders.

This approach enabled the identification of the social and cultural logics underlying the observed behaviors, highlighting discrepancies among declared knowledge, risk perceptions, and actual practices. The qualitative results were thus used to contextualize and interpret the quantitative data, particularly the observed divergences across the dimensions of KAP, from a socio-anthropological perspective [23], [24].

### 2.9 Ethical considerations

This study was conducted in accordance with the Declaration of Helsinki and relevant national regulations governing research involving human participants in Benin. Ethical approval was obtained from the Research Ethics Committee of the “Institut des Sciences Biomédicales Appliquées (CER-ISBA)”, Benin (Favorable Ethical Decision No. 246, 2026), prior to data collection. All participants received information about the study objectives and procedures and provided informed consent before participation. Participation was voluntary, and participants could decline or withdraw from the study without consequence. Confidentiality was maintained throughout data collection, data management, and analysis. Participants' data were handled without disclosure of personally identifying information and were used solely for research purposes. Administrative authorization for field activities was also obtained from the Ministry of Agriculture, Livestock and Fisheries of Benin, with approval and facilitation from the relevant authorities of Agricultural Development Pole 4 (ATDA4) and the Departmental Directorates of Agriculture, Livestock and Fisheries (DDAEP).

## 3 Results

### 3.1 Socio-demographic and professional profile of the actors surveyed

As shown in Table 3, the 164 livestock farmers surveyed came primarily from Donga and Borgou, confirming the strong representation of the major pastoral basins of these two departments, followed by Collines and Zou. At the municipal level, Djougou alone accounted for a third of the sample, ahead of Tchaourou, Nikki, Bassila, Savalou, Djidja, and Dassa. Among veterinary staff, the largest share came from Borgou and Donga, with a high concentration in Tchaourou and Djougou. Butchers, although few in number, were distributed across the four departments of the PDA_4_, with a slight predominance from Borgou.

**Table 3:** Profile of the socio-demographic and professional characteristics of the actors surveyed in the PDA_4_.

Variable	Livestock farmers (*n* = 164)	Veterinarians (*n* = 36)	Butchers (*n* = 11)
Men	92.7%	88.9%	100%
Middle age	44.9 ± 11.3	37.9 ± 8.9	41.5 ± 12.7
Uneducated	76.2%	2.8% primary only; 63.9% higher	63.6%
Married	100%	88.9%	100%
Muslims	98.2%	69.4%	90.9%
Peuhl/Foulani	98.2%	58.3%	72.7%
>10 years of seniority	84.7%	55.6%	90.9%

Socially, livestock farmers were predominantly male, middle-aged, married, Muslim, and Fulani, with a particularly low level of formal education (Table 3). The family profile also showed a substantial domestic burden, with 58.6% having more than five children.

From an economic and professional standpoint, nearly half of the livestock farmers reported having virtually no monthly income (47.6%), and most had more than 10 years of experience. Livestock farming was overwhelmingly traditional, based on natural grazing (93.3%), with only a small proportion practicing semi-intensive or intensive farming. The average herd size was 60.5 ± 64.2 head, with herds of 26 to 50 head most represented, suggesting an intermediate production base without excluding a subgroup of large-scale farmers.

The veterinarian group comprised 36 respondents. They were predominantly male and middle-aged but, unlike livestock farmers, had higher education levels and more stable monthly incomes. The majority were married and Muslim, but their ethnic backgrounds were more diverse than those of the livestock farmers. More than half had over 10 years of professional experience.

The butchers numbered 11, were all men, and generally had extensive professional experience. Their geographical distribution covered the Donga, Collines, Borgou, and Zou regions, with a presence in Djougou, Dassa, Tchaourou, Nikki, and Djidja. Most were married and Muslim, while their level of formal education remained relatively low. Their seniority was very high, with the majority having worked for more than 10 years.

Table 3 summarizes the socio-demographic and professional characteristics of the livestock farmers, veterinarians, and butchers included in the study.

### 3.2 Watering system and motivations for choosing the activity

**Fig. 2:**
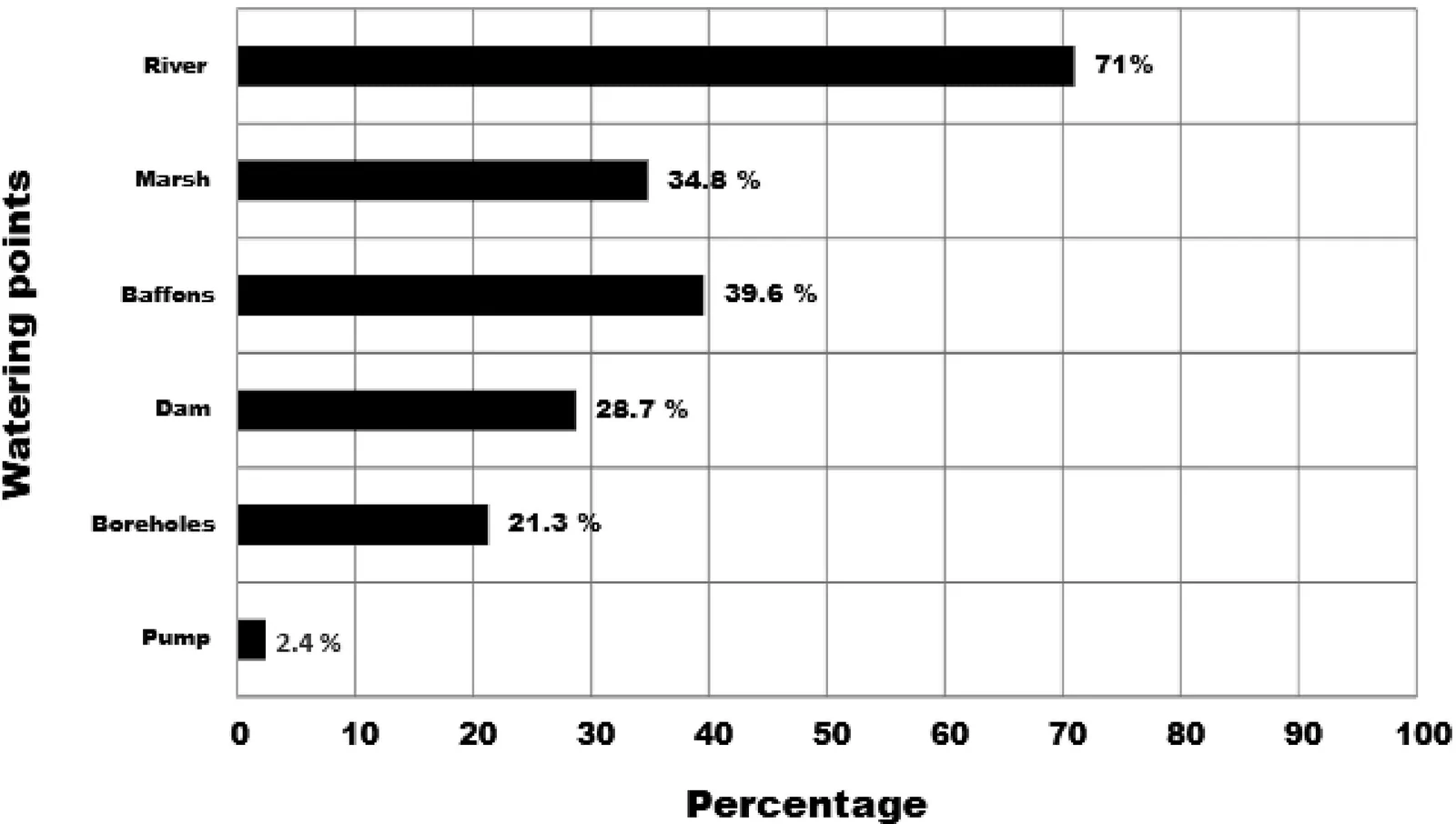
Main types of watering points used by livestock farmers.

Analysis of watering points shows in Fig. 2 a strong dependence on surface water, dominated by natural water points. Rivers were by far the most used water source (71.3%), well ahead of the other categories detailed in Fig. 2, while engineered water points such as boreholes and pumps remained marginal.

Consistent with this result, the qualitative component describes these places as areas of high animal concentration, particularly in the dry season, when water becomes scarce, and several herds gather around the same water resources.

**Fig. 3:**
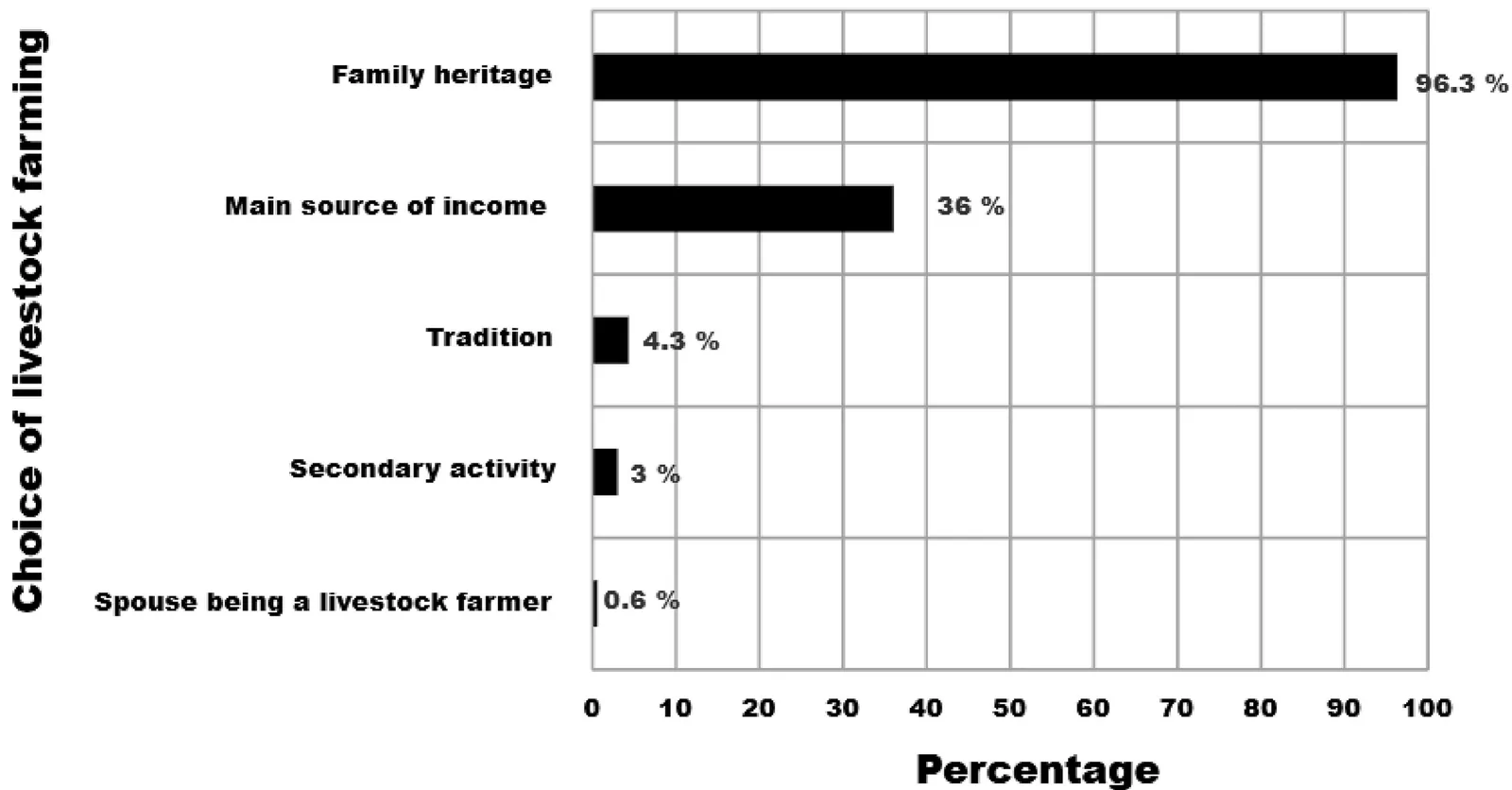
Motivations for choosing livestock farming as an activity.

Furthermore, the motivations for choosing livestock farming were overwhelmingly dominated by family heritage, far ahead of the main source of income, tradition, secondary activity, or having a spouse who is a livestock farmer. This data confirms that cattle farming in the PDA_4_ is deeply rooted in family and cultural transmission (Fig. 3).

### 3.3 KAP scores and correlations of livestock farmers

The farmers' KAP scores revealed a highly disassociated profile, as shown in Table 4. The mean knowledge score reflected an overall low level of understanding and formal knowledge of bTB and its transmission mechanisms. Conversely, attitude scores suggested that farmers generally expressed a favorable disposition toward recognizing the problem or considering it important. Practices, however, remained limited. These results show a clear dissociation between stated perception and actual implementation of preventive practices. The Shapiro-Wilk tests indicated an asymmetry in the three distributions, justifying the use of Spearman's rank correlation coefficient.

**Table 4:** Distribution of knowledge, attitudes, and practices scores among livestock farmers, veterinarians, and butchers.

Group	Dimension	Average	Median	Interpretation
Livestock farmers	Knowledge	41.6%	42.9%	Weak
	Attitudes	80.0%	80.0%	High
	Practices	51.5%	44.4%	Moderate to low
Veterinarians	Knowledge	80.0%	85.7%	High
	Attitudes	42.2%	40.0%	Weak
	Practices	44.8%	50.0%	Moderate to low
Butchers	Knowledge	63.6%	80.0%	Moderate to low
	Attitudes	97.0%	100.0%	High
	Practices	69.1%	60.0%	High

The correlational analysis summarized in Table 5 showed a moderately strong, highly significant positive correlation between knowledge and attitudes (ρ = 0.42; *p* < 0.001), indicating that higher knowledge scores tended to be associated with more favorable attitude scores. In contrast, the correlation between knowledge and practices was extremely weak and not statistically significant, indicating no detectable association between these dimensions in this sample. Finally, attitudes were weakly but significantly correlated with practices (ρ = 0.19; *p* = 0.018), suggesting that a better perception of risk could have a partial, but limited, effect on behavior.

**Table 5:** Correlations between knowledge, attitudes, and practices among livestock farmers.

Group	Association	ρ	*p*-value
Livestock farmers	K Score - A Score	0.42	<0.001
	K Score - P Score	0.014	0.86
	A Score - P Score	0.19	0.018
Veterinarians	K Score - A Score	−0.39	0.022
	K Score - P Score	0.059	0.74
	A Score - P Score	−0.004	0.98
Butchers	K Score - A Score	0.50	0.12
	K Score - P score	−0.22	0.51
	A Score - P Score	−0.35	0.30

### 3.4 KAP scores and correlations for veterinary staff

Table 4 shows that veterinary staff achieved the highest knowledge score, as expected given their training and technical responsibilities. However, their attitudes remained low, and their practices were only moderate.

Spearman's correlation analysis (Table 5) showed a moderate, statistically significant negative relationship between knowledge and attitudes (ρ = −0.39; *p* = 0.02). This suggests that veterinarians with the highest levels of knowledge expressed less favorable attitudes toward bTB management. No significant correlation was observed between knowledge and practices or between attitudes and practices, indicating that professional behaviors are not directly determined by declared knowledge or perceptions.

### 3.5 KAP scores and correlations for butchers

Among butchers, the average scores reflected intermediate knowledge, very favorable attitudes, and relatively better practices, as shown in Table 4.

No statistically significant correlations were observed among Knowledge, Attitudes, and Practices (KAP) scores, although knowledge and attitudes showed a moderate positive correlation. Refer to Table 5 for details. However, interpret these results cautiously due to the small sample size.

### 3.6 Qualitative results

Qualitative data provided essential insights into the discrepancies observed in quantitative scores (Table 6). Among livestock farmers, the disease was often recognized through signs such as coughing and weight loss, but could be mistaken for a simple respiratory illness. Local names such as “Èrè,” “Rere,” “Doyru,” “Tokotoko,” or “Hèrrè” indicate the disease's presence in pastoral parlance, though this does not necessarily imply an understanding of its zoonotic nature. Transmission from animals to humans remained poorly understood. However, many farmers did not clearly recognize the disease's zoonotic nature, and only some were aware that consuming raw milk or close contact could transmit it to humans. Veterinarians, on the other hand, described a contagious disease often more visible at slaughter than within the herd.

**Table 6:** Main qualitative results.

Theme	Key findings
Local knowledge	Common names for the disease, partial recognition of the signs
Representations	Confusion with other illnesses, sometimes a mystical interpretation
Dietary practices	Frequent consumption of raw milk
Care	Initial recourse to medicinal plants
Management of sick animals	Sale, donation, or informal slaughter possible.
Environment	Sharing of water points, stagnant water, and mixing of herds
Communication	Importance of local radio stations, Fulani leaders, and Departmental Union of Professional Livestock Breeders Organizations (UDOPER)

Risky practices were deeply entrenched. Raw milk consumption was described as almost systematic in several communes. The use of medicinal plants often preceded calling a veterinarian, delayed veterinary care, the informal sale or slaughter of sick animals, and poor hygiene or the use of protective equipment. Shared watering points, proximity between animals and living spaces, and the mixing of herds during the dry season are described as major exposure factors.

The environment played a crucial role. Shared watering points, stagnant pools, and herd concentration during the dry season facilitated contact between herds and environmental contamination. Finally, community leaders and local radio stations, particularly in certain municipalities, were described as potentially more effective channels for raising awareness than external technical messages.

The qualitative results confirm that the observed practices do not stem solely from a lack of knowledge but from a structured socio-cultural system in which pastoral norms, economic constraints, and symbolic representations of illness shape behavior. These observations align with research in the socio-anthropology of health, which demonstrates that health behaviors are largely structured by social norms, economic constraints, and local systems of representation [23], [24], [25].

## 4 Discussion

### 4.1 Socio-demographic and territorial determinants of bTB risk

This survey indicates that bTB transmission in the Agricultural Development Pole 4 (PDA_4_) region cannot be explained solely by diagnostic or veterinary shortcomings. Instead, it reflects a broader socio-ecological system where social, occupational, territorial, and environmental factors interact. Livestock farmers form a relatively uniform social group, mainly Fulani, Muslim, married, and with limited education, who have long been involved in traditional pastoralism. Conversely, veterinarians tend to be younger, better educated, and more integrated into institutions, while butchers hold an intermediate position with extensive experience but limited formal education. These differences are significant because they directly shape perceptions, interpretations, priorities, and management of bTB within communities.

Among farmers, low knowledge scores appear closely linked to this social structure. The dominance of inherited pastoral routines, family-based livestock management, and deep-rooted cultural norms may partly explain why biomedical knowledge remains limited despite generally positive attitudes toward disease prevention. Notably, 76.2% of farmers lack formal education, which does not necessarily mean they are unaware of diseases; rather, it reflects the dominance of empirical and local knowledge over biomedical understanding. Qualitative data support this: farmers often identified the disease using local terminology and visible clinical signs, with limited understanding of its zoonotic aspect.

The spatial distribution of participants further emphasizes the importance of geographically contextualized analysis. Donga and Borgou, especially Djougou and Tchaourou, are major pastoral basins where animal movement, transhumance, shared watering points, and herd mixing significantly shape exposure and risk perceptions. Conversely, the Collines and Zou regions provided insights into slaughter-related activities, traditional beliefs, and localized perceptions of disease. This territorial variation indicates that the PDA4 region is not epidemiologically uniform, as local pastoral systems, access to water, slaughter practices, and sociocultural norms vary considerably across municipalities.

The observed social homogeneity among livestock farmers also suggests that collective norms and identity-based behaviors reinforce risky practices. In pastoral communities, practices such as raw milk consumption, traditional treatments, and informal animal care are not just individual choices but are embedded in family traditions, social structures, and pastoral identity. Similar findings have been observed in several pastoral systems across sub-Saharan Africa, where sociocultural and environmental factors influence zoonotic risk perception more than biomedical knowledge [14], [35]_._

These territorial and socio-demographic patterns echo findings from other pastoral and agro-pastoral settings in the region. Among smallholder livestock farmers in Rwanda, low formal education and reliance on traditional husbandry practices were similarly associated with limited biomedical knowledge of zoonotic diseases despite a generally favorable disposition toward prevention [36]. Likewise, a community-based KAP survey on bovine tuberculosis in Yemalogi Welel district, Ethiopia, reported that community-level understanding of the disease remained closely tied to local terminology and visible clinical signs rather than to formal knowledge of its zoonotic transmission, mirroring the pattern observed among farmers in the present study [37]. Taken together, these convergent findings across distinct sub-Saharan pastoral contexts suggest that the socio-territorial determinants of bTB-related knowledge identified in PDA4 are not idiosyncratic to Benin but reflect a broader regional pattern that control strategies should account for.

### 4.2 Knowledge, attitude, practice gaps, and structural determinants among actors

A key finding of this survey was the ongoing disconnect among various actor groups in KAP. While livestock farmers showed a strong positive link between knowledge and attitudes, the connection between attitudes and protective behaviors was weak, indicating that awareness alone may not change behavior. Similar patterns have been observed in pastoral communities across Africa and other low- and middle-income countries, where risky behaviors such as consuming raw milk, informal slaughtering, or handling animals without protection persist despite some awareness of zoonotic risks [36], [37], [38]. These results align with Launiala [23], who noted that KAP surveys do not necessarily predict actual behavior, especially when social norms, economic vulnerability, and structural factors heavily influence daily practices.

Economic conditions significantly influence this knowledge-behavior gap. Nearly half of livestock farmers (47.6%) reported unstable monthly incomes, and 16.3% earned less than €76.22 per month, with many living on low incomes. In such circumstances, behaviors like delaying veterinary visits, using traditional remedies, or selling sick animals may be survival strategies rather than negligence or denial. These insights suggest that health behaviors should be viewed within broader socio-economic and structural contexts, rather than focusing solely on knowledge. This approach avoids moral judgments of poor practices and considers the economic realities faced by pastoral households.

Among veterinarians, the observed negative correlation between knowledge and attitudes likely reflects professional realism rather than lack of commitment. More knowledgeable veterinarians might be more aware of operational challenges in controlling bTB, such as limited resources, institutional support, low community compliance with prevention measures, and the practical difficulties of implementing surveillance in pastoral areas. Previous research in veterinary public health and animal health economics also shows that professional practices are shaped more by institutional and logistical constraints than by technical knowledge alone [16].

Among butchers, more positive attitudes and practices may be linked to their direct occupational exposure to carcasses, lesions, and tissues during slaughter and meat processing. Their practical experience may help them recognize suspicious lesions and zoonotic risks, even without formal biomedical training. However, these findings should be interpreted with caution due to the small sample size (*n* = 11), which limits statistical power. Several studies in sub-Saharan Africa report ongoing occupational exposure to *M. bovis* among slaughterhouse workers and meat handlers [15]. Recent WOAH guidelines also identify butchers and abattoir workers as high-risk groups at the livestock-human interface [17]^.^ Therefore, good practices alone may not fully mitigate occupational risks in slaughterhouse settings.

Qualitative data emphasize the sociocultural factors affecting disease spread. Practices like consuming raw milk, using medicinal plants, and the informal sale or slaughter of sick animals are deeply embedded in local trust, cultural identity, and economic needs. These findings suggest that disease control methods that rely solely on top-down technical messages are unlikely to produce lasting behavioral change. Instead, interventions should incorporate local languages, engage community leaders, utilize radio outreach, and include health education tailored to cultural contexts. Overall, these insights highlight the need for integrated, context-aware strategies to manage zoonotic diseases within pastoral socio-ecological systems.

### 4.3 Environmental interface and one health implications

Another key insight from this survey highlights the vital role of watering points in pastoral systems. Rivers, marshes, dams, and seasonal water sources primarily support livestock watering and often serve as hubs for herd congregation, animal mixing, and organic contamination, especially during dry periods. These environmental interfaces likely enable indirect interactions among animals, humans, and contaminated media such as water, soil, and forage. These findings align with the One Health framework used in this study, which treats the environment as an active factor in *M. bovis* transmission rather than a passive background element. In pastoral systems with high animal mobility and shared resources, environmental contamination can sustain transmission across different species [13]. Therefore, integrating environmental, veterinary, and human health perspectives is crucial to enhance surveillance and develop effective, locally tailored control measures. This environmental interface pathway has been documented elsewhere in West Africa: shared watering points and communal grazing areas were similarly identified as key risk factors for *M. bovis* transmission and persistence among urban and peri-urban cattle herds in Niger [10], and dry-season concentration around limited water resources has more broadly been described as a driver of both intra- and inter-herd transmission of zoonotic *M. bovis* in extensive pastoral systems [11]. This convergence across settings reinforces the relevance of the environmental interface as a priority target for One Health surveillance in PDA4.

### 4.4 Implications for action, strengths, and limitations

This study indicates that uniform awareness strategies are probably ineffective in pastoral areas with high social, territorial, and occupational diversity. For livestock farmers, prevention efforts should incorporate culturally tailored communication methods that engage community leaders, use local languages, leverage radio, and draw on familiar forms of disease knowledge. For veterinarians, strengthening operational capacity, surveillance tools, and institutional support may be more vital than technical training alone. For butchers and slaughterhouse workers, targeted measures on occupational hygiene, carcass inspection, and safe handling are crucial. More broadly, interventions must take into account the realities of the Donga, Borgou, Collines, and Zou regions, as their social and professional structures are not perfectly aligned.

A key strength of this survey is its mixed-methods approach, combining quantitative and qualitative data to understand better behavioral, social, and environmental factors influencing bTB risk through a One Health lens. Nonetheless, some limitations are worth noting. The small number of butchers in the sample weakens the correlation analysis. Also, as a cross-sectional study, it cannot establish causality between socioeconomic factors and health practices.

Overall, these findings suggest that strategies for preventing and controlling bTB in pastoral systems should extend beyond purely biomedical methods by integrating social, economic, territorial, and environmental factors that influence disease spread. The study emphasizes the complex interactions at the human-animal-environment interface and underscores the importance of integrated One Health strategies for managing zoonotic tuberculosis in West Africa.

## 5 Conclusion

Considering socio-demographic nuances greatly enriches the interpretation of the KAP results in the PDA_4_. Among livestock farmers, limited knowledge and insufficient practices reflect a social profile characterized by low levels of education, cultural homogeneity, long tenure, and the dominance of traditional livestock farming. Among veterinarians, the contrast between high knowledge and low attitudes may reflect limitations in the control system rather than a lack of commitment. Among butchers, extensive professional experience and direct exposure to carcasses may explain a more favorable profile, despite the small workforce.

In summary, the data show that bTB in PDA_4_ cannot be understood or combated solely through biomedical information. It must be addressed as a situated, socially structured, professionally differentiated, and ecologically sustained problem. These findings are consistent with the One Health approach, which emphasizes integrating social, environmental, and institutional dimensions into zoonotic disease management.

## CRediT authorship contribution statement

**Nongande Pioulasse Bernice Houecande:** Writing – original draft, Investigation, Funding acquisition, Formal analysis, Data curation, Conceptualization. **Tamégnon Victorien Dougnon:** Writing – review & editing, Supervision, Resources, Project administration, Funding acquisition, Conceptualization. **Roch Houngnihin:** Writing – review & editing, Formal analysis. **Souaïbou Farougou:** Writing – review & editing, Conceptualization. **Kadoéito Cyrille Boko:** Writing – review & editing, Supervision, Project administration, Methodology, Funding acquisition, Conceptualization. **Claude Saegerman:** Writing – review & editing, Validation, Supervision, Methodology, Data curation, Conceptualization.

## Declaration of competing interest

The authors declare that they have no known competing financial interests or personal relationships that could have appeared to influence the work reported in this paper. The authors are really grateful to the Programme Belge d'Appui Institutionnel between the University of Abomey-Calavi (Benin) and the Académie de Recherche et d'Enseignement Supérieur (ARES) from Belgium.

## Data Availability

Data will be made available on request.
